# Comparison between fluoropyrimidine-hepatic arterial infusion and systemic chemotherapy for unresectable liver metastases: A protocol for systematic review and meta-analysis based on 16 observational studies

**DOI:** 10.1097/MD.0000000000027483

**Published:** 2021-10-15

**Authors:** Jianmeng Zhao, Yuenan Zheng, Tao Liu, Jinzhe Chang, Hongqing Shan, Ke Cong

**Affiliations:** Department of General Surgery, Guangrao County People's Hospital, Guangrao, China.

**Keywords:** hepatic arterial infusion, liver cancer, liver metastases, systemic chemotherapy

## Abstract

**Background:**

The benefit of loco-regional treatments such as hepatic arterial infusion (HAI) in terms of survival and response rate is unclear. The aim of this work is to quantitatively summarize the results of both randomized controlled trials (RCTs) and non-randomized studies of interventions (NRSIs) comparing fluoropyrimidine-HAI (F-HAI) to systemic chemotherapy (SCT) for the treatment of colorectal liver metastases (CRLMs).

**Methods:**

We searched the Cochrane Library, PubMed, EMBASE, and Web of Science up to July 1, 2021. The outcome measures were tumor response rate and overall survival (OS). Both RCTs and NRSIs comparing HAI to SCT for patients with unresectable CRLMs were included. The outcome measures were tumor response rate and OS. Two reviewers assessed trial quality and extracted data independently. All statistical analyses were performed using standard statistical procedures provided in Review Manager 5.2.

**Results:**

A total of 16 studies including 11 RCTs and 5 NRSIs were identified for the present meta-analysis. Nine RCTs compared F-HAI to SCT for patients with unresectable CRLMs and the pooled result indicated that patients who received F-HAI experienced more than twofold response rate than SCT, with a pooled risk ratio of 2.10 (95%CI 1.59–2.79; *P* < .00001). In addition, the pooled result based on RCTs showed that F-HAI had a significant benefit regarding OS, with a pooled HR of 0.83 (95% CI 0.70–0.99; *P* = .04). Similarly, the benefit of F-HAI in terms of OS was also observed in the results of NRSIs.

**Conclusions:**

Our results indicated that the F-HAI regimen had a greater tumor response rate and survival advantage than SCT for patients with unresectable CRLMs. Future propensity score-matched analyses with a large sample size should be conducted to support the evidence of our results based on RCTs and NRSIs.

## 1 Introduction

Nearly 50% of colorectal cancer (CRC) patients will develop liver metastases during the course of their disease, with half having hepatic metastases at the time of primary diagnosis and another half developing metachronous disease.^[1]^ Furthermore, more than 50% of patients who die of CRC have liver metastases at autopsy, and the majority of these patients die as a result of their metastatic liver disease. Surgical resection represents the standard treatment of resectable disease and results in 5-year overall survival (OS) rates of 20% to 40%.^[1,2]^

Unfortunately, only 20% of patients with colorectal liver metastases (CRLMs) present with liver-confined resectable disease and/or are a candidate for major surgical operation (depending on comorbidities). At present, unresectable liver-confined metastatic disease from CRC is a challenging therapeutic issue: the prognosis of these patients is dismal (median survival: 6–18 months), as no curative option currently exists.^[1,3]^

Unresectable hepatic metastatic disease from CRC is generally associated with a dismal prognosis.^[1–3]^ Despite the improvements achieved with modern SCT regimens (eg, those combining 5-fluorouracil with irinotecan or oxaliplatin), the median OS time of these patients does not exceed 18 to 20 months, with a 5-year OS rate of close to 0% (ie, there are virtually no long-term survivors). In light of these disappointing results, novel therapeutic strategies are eagerly needed.

Although locoregional treatments such as hepatic arterial infusion (HAI) claim the advantage of delivering higher doses of anticancer agents directly into the metastatic organ (as compared to systemic chemotherapy [SCT]), the benefit in terms of OS is undefined. According to 2 meta-analyses published more than 10 years ago on the results of 6 of 7 available RCT, fluoropyrimidine-HAI (F-HAI) appears to provide a small but significant survival advantage over fluoropyrimidine-based SCT.^[4,5]^ However, since then, several additional RCTs and non-randomized studies of interventions (NRSIs) have been carried out. As the findings of these studies are conflicting, it appears important to quantitatively summarize the available evidence in this field. Therefore, we quantitatively summarized the results of both RCTs and NRSIs comparing F-HAI to SCT for the treatment of unresectable CRLMs.

## 2 Methods and materials

### 2.1 Criteria for considering studies

We included studies if: (1) Adults with unresectable CRC liver metastatic disease who were randomized to receive HAI or SCT; (2) HAI (experimental treatment): HAI of a fluoropyrimidine compound (either 5-fluorouracil or floxuridine [FUDR]); SCT (control arm): SCT (any type); (3) the outcomes including overall tumor response rate and OS; and (4) both RCTs and NRSIs comparing F-HAI to SCT for the treatment of unresectable CRLMs.

Studies will be excluded if they meet the following criteria: (1) patients suffered other tumors or metastasis that may influence our outcomes; (2) experimental trials on animals or non-human studies; (3) studies not conducted in the adult population; (4) abstracts, letters, editorials, expert opinions, reviews, and case reports were excluded; and (5) studies without sufficient data or did not meet our including criteria were excluded.

### 2.2 Search strategy

We searched the Cochrane Library, PubMed, EMBASE, and Web of Science up to July 1, 2021. Our strategy was based on combinations of medical subject headings (MeSH) and the keywords: “colorectal,” “colon,” “rectal,” “cancer,” “carcinoma,” “liver,” “hepatic,” “metastasis,” “metastases,” “metastatic,” “hepatic arterial infusion,” “intra-arterial,” “infusion,” “HAI,” “chemotherapy,” and “systemic.” Two assessors independently screened the titles and abstracts of each study. Once relevant studies became certain, the full texts were obtained for further evaluation. Other related references we read were also searched online for full texts and assessment, once any of them meet our inclusion criteria, they will also be included in this meta-analysis.

### 2.3 Quality assessment and data extraction

Two reviewers assessed the quality of each RCT using the previously validated 5-point Jadad scale.^[6]^ Studies with scores of 1–2 were considered low quality; scores of 3 or more were considered high quality. In addition, the risk of bias for each study and the risk of bias across all studies were evaluated and shown with figures generated by RevMan 5.2 software.^[7]^ For the quality of NRSIs, the 9-star Newcastle–Ottawa Scale^[8]^ was used and we considered studies with NOS score more than 6 as high quality. The evaluated total scores of each study were displayed in Tables 1 and 2.

**Table 1:** The characteristics of included RCTs for the present systematic review and meta-analysis.

		Sample size (pts)		Patients treated		High tumor burden (%)					
Study (author/year)	Country	F-HAI	SCT	F-HAI	SCT	F-HAI	SCT	Response rate	Median survival	Outcomes	Jadad score
Allen-Mersh et al 1994	United Kingdom	51	49	96%	20%	27%	22%	NR	13.5 mo7.5 mo	Quality of life and survival	2
Chang et al 1987	United States	32	32	66%	92%	81%	81%	62%17%	17 mo12 mo	Response amd, survival rate, toxicity, side effects	3
Hohn et al 1989	United States	67	76	75%	86%	46%41%	41%	42%9%	16.5 mo15.8 mo	Hepatic response rate, time to hepatic progression, and toxicity	3
Kemeny et al 1987	United States	48	51	94%	94%	50%	65%	53%21%	17 mo12 mo	Response rate, Toxicity and survival	2
Kemeny et al 2006	United States	68	67	87	87	71%	70%	47%24%	24.4 mo20 mo	Overall survival, response rates, THP, TEP, quality-of-life, toxicity	4
Kerr et al 2003	United Kingdom	145	145	66	87	50%	50%	22%19%	14.7 mo14.8 mo	Overall survival, toxicity, progression-free survival.	4
Lorenz et al 2000	Germany	54	57	69	91	69%	67%	43%27%	18.7 mo17.6 mo	TDP, survival times, AEs	3
Martin et al 1990	United States	36	33	79%	83%	72%	79%	48%21%	12.6 mo10.5 mo	Tumor response, THP, overall survival and progression	2
Peng et al 2021	China	62	196	62	62	NR	NR	25%; 28.9%	14.0 mo10.8 mo	Objective response rate and overall survival	1
Rougier et al 1992	France	81	82	87%	50%	41%	44%	41%9%	15 mo11 mo	Survival, AEs	1
Wagman et al 1990	United States	31	10	100%	100%	100%	100%	55%20%	13.8 mo11.6 mo	Median TTF and survival	2

**Table 2:** The characteristics of included NRSIs for the present systematic review and meta-analysis.

Study (author/year)	Country	Sample size (pts)	Age (median, range) (year)	Therapy	Regimens	Follow-up time (median, range)	Outcomes	NOS scale
Bolton et al 2012	United States	49	61.5 (25–75)61.0 (47–75)	HAI FUDR + I.V. 5-FU/LV	0.2 mg/kg/day over 14 days for a total of 42-week treatments. 5-FU 425 mg/m^2^/day and LV 20 mg/m^2^ /day daily for 5 days for a total of 4 5-day treatment	Every 3 mo × 4 and then every 6 mo to 5.5 yrs	Recurrence, DFS, Survival	8
Dhir et al 2017	United States	86	59 (51–69)	HAI + modern SCT	0.12 mg/(kg/day) 9 kg 9 pump volume/flow rate in a 4-week cycle; HAI is started on day 1 of each cycle. SCT is started at least 2 weeks after HAI pump placement	NR	Survival, demographic and treatment characteristics	7
House et al 2011	United States	250	55 (28–80)61 (25–84)	adjuvant HAI-FUDR/Dex + SCT	Adjuvant systemic chemotherapy including FU, leukovorin (LV) plus oxaliplatin or irinotecan in addition to HAI-FUDR/Dex	43 mo (0.5–92)	3- and 5-yr DSS, survival, RFS	8
Li et al 2014	China	35	60 (19–75)	SCT plus HAI FUDR	0.12 mg/kg/day when combined with systemic m-FOLFOX6.	18.45 mo (2.77–61.83)	Response and survival	7
Samaras et al 2011	Switzerland	23	60.3 (39.7–76.9)	FUDR-HAI + SCT	FUDR-HAI was administered every 28 days as a 14-day infusion at 0.12 mg/kg 9 30 divided by flow rate. SCT was administered FOLFOX-like regimen with oxaliplatin 100 mg/m^2^ on day 1, leucovorin 400 mg/m^2^ on day 1, and 5-fluorouracil 1200 mg/m^2^ over 46 h.	At least every 2 wks	Response and survival, risk factor, complications, AEs, and toxicities	8

Data for the comparative analysis of F-HAI versus SCT for unresectable CRLMs were extracted independently by 2 reviewers, and disagreement was resolved through discussion. The extracted contents, including first authors, published years, country, sample size, interventions, outcomes, and quality score of each study, were displayed using a standardized form. Data collected were input into RevMan 5.2 software for analysis.^[7]^

### 2.4 Statistical analysis

The data of comparable outcomes between F-HAI and SCT for unresectable CRLMs were combined-analyzed, using the standard statistical procedures provided in RevMan 5.2.^[7]^ Dichotomous data were measured with risk ratio (RR) or odds ratio (OR). Survival outcomes were measured with a hazard ratio (HR). The heterogeneity between studies was evaluated by the chi-square-based Q statistical test,^[9]^ with *P*_*h*_ value and *I*^2^ statistic, ranging from 0% to 100%, to quantify the effect of heterogeneity. *P*_*h*_ ≤ .10 was deemed to represent significant heterogeneity,^[10]^ and pooled estimates were estimated using a random-effect model (the DerSimonian and Laird method^[11]^). On the contrary, if statistical study heterogeneity was not observed (*P*_*h*_ > .10), a fixed-effects model (the Mantel–Haenszel method^[12]^) was used. The effects of outcome measures were considered to be statistically significant if pooled RRs/HRs with 95% CI did not overlap with 1. In addition, OS was further analyzed with subgroup analysis according to different characteristics. All statistical analyses were performed using standard statistical procedures provided in RevMan 5.2.^[7]^

This work has been reported in line with Preferred Reporting Items for Systematic Reviews and Meta-Analyses^[13]^ and Assessing the methodological quality of systematic reviews Guidelines.^[14]^ The present study was approved by the Ethics Committee of Guangrao County People's Hospital.

## 3 Results

### 3.1 Included studies, study characteristics, and quality assessment

**Figure 1:**
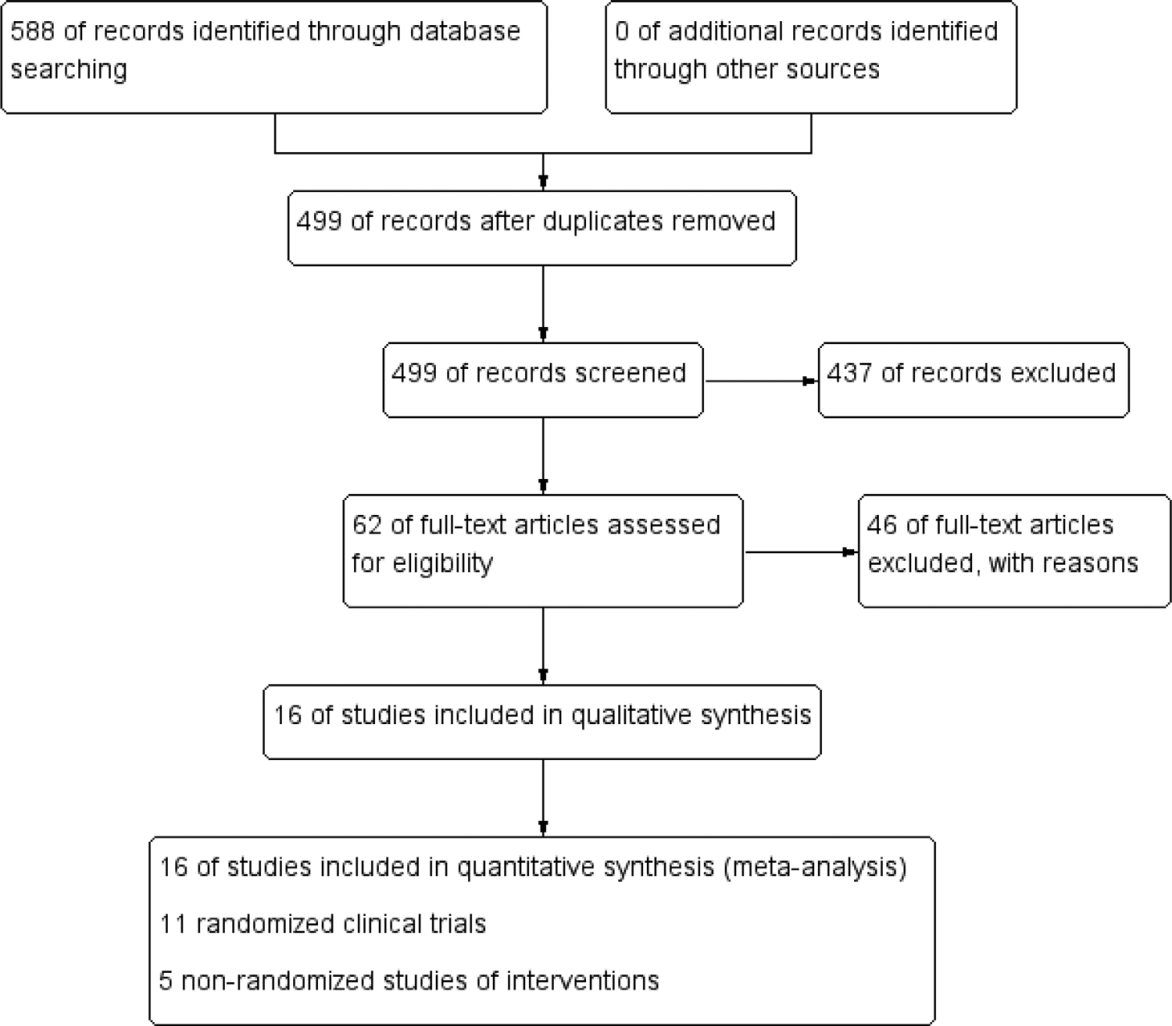
Flow diagram following the PRISMA template of the search strategy. PRISMA = Preferred Reporting Items for Systematic Reviews and Meta-Analyses.

In total, 588 studies were initially identified; after duplicates were removed, the titles and abstracts of 499 studies were screened. Of these, 438 studies were excluded, and the full texts of the remaining 62 studies were obtained for further evaluation. After reading the full texts, 46 studies were excluded for various reasons such that, ultimately, 16 studies^[15–30]^ (N = 1916 participants) were included in this meta-analysis (Fig. 1). Among the studies, the sample size ranged from 23 to 290 patients.^[23,28]^ Nine studies were from the United States.^[16–22,26,29]^ Eleven studies with 1471 patients were RCTs (Table 1),^[15,17,19,21–23,25–27,29,30]^ and 5 studies with 443 patients were NRSIs (Table 2).^[16,18,20,24,28]^

**Figure 2:**
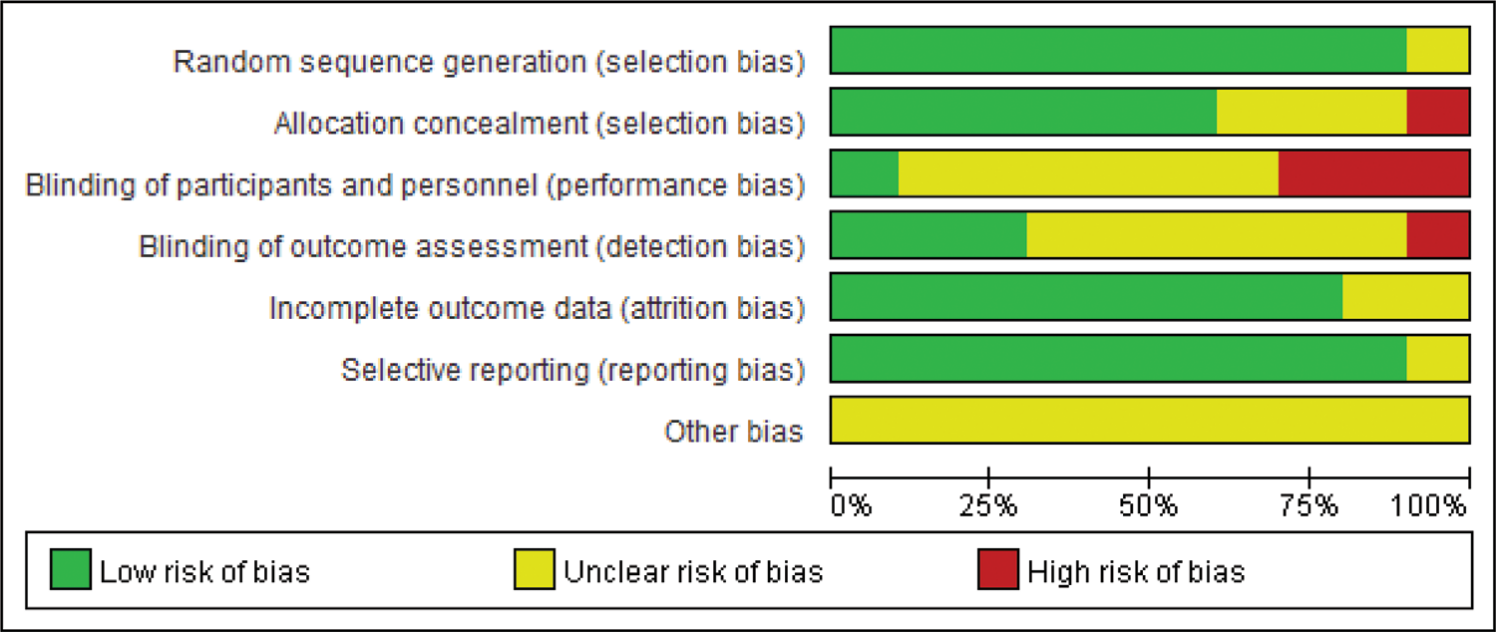
Risk of bias graph: review authors’ judgments about each risk of bias item presented as percentages across all included studies.

**Figure 3:**
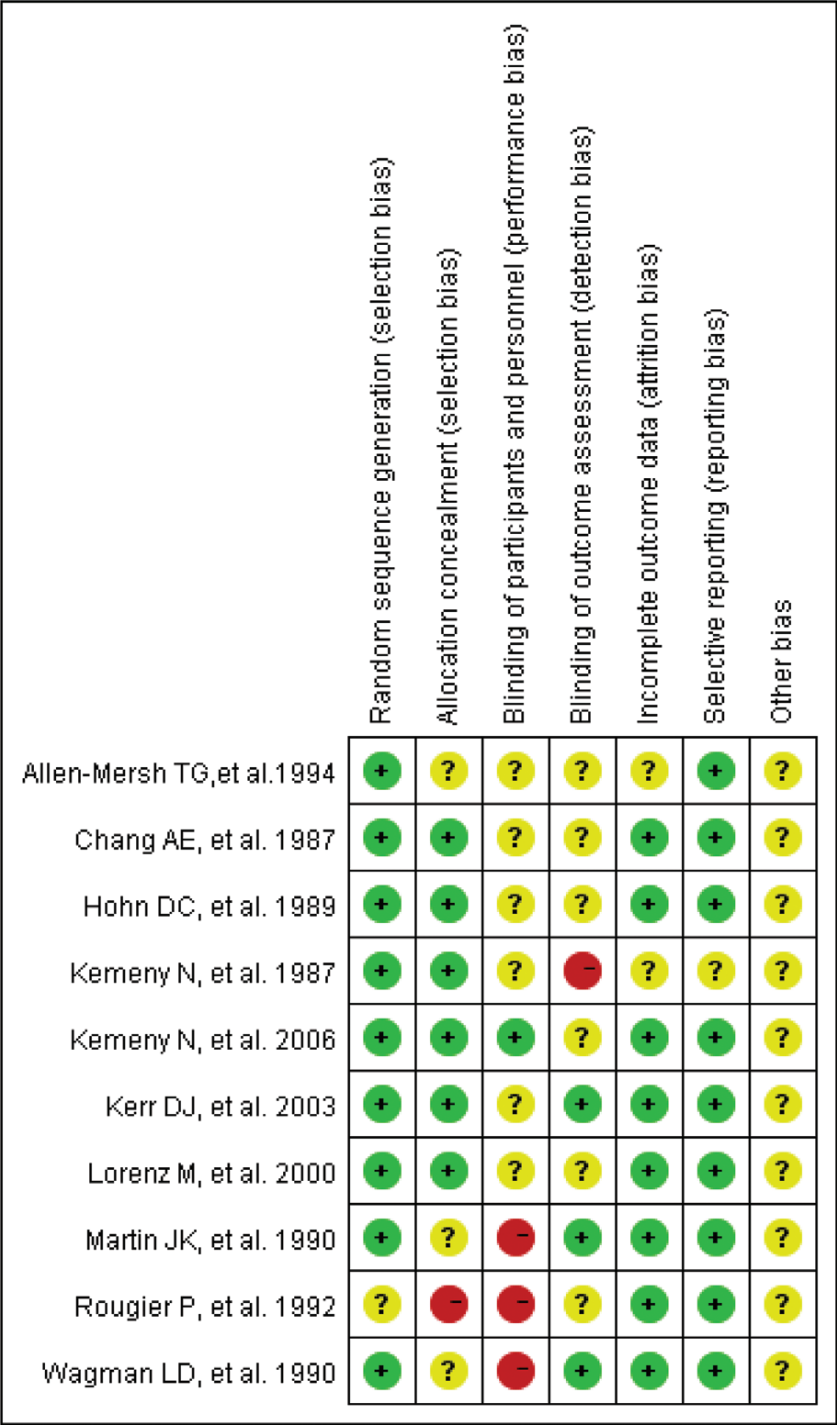
Risk of bias summary: review authors’ judgments about each risk of bias item for each included study.

According to our definitions, 5 of 10 RCTs were evaluated with a Jaded score ≥3 and half of the studies experienced high quality. For NRSI, all of the 5 included studies for pooled analysis experienced a NOS score ≥6 and were considered as good quality. Graphs showing the risk of bias of RCTs were then generated. The overall risk of bias for each RCT is presented as a percentage relative to all included studies in Figure 2, and the risk of individual types of bias is displayed in Figure 3. The risk of bias graphs for the RCTs indicated generally good methodological quality, mainly in terms of selection and reporting biases. However, there was a high risk of performance bias in some studies. Unclear risk of bias was mainly seen in terms of performance, detection, and “other” biases.

### 3.2 Comparison of response rate between F-HAI and SCT

A total of 10 RCTs reported raw data on overall response rates, which included complete (disease disappearance) and partial (disease shrinkage >50%) tumor responses. Singularly taken, 6 RCT reported a significantly higher response rate in the HAI arm compared with the SCT arm whereas, in 3 RCT, no significant difference was observed.

**Figure 4:**
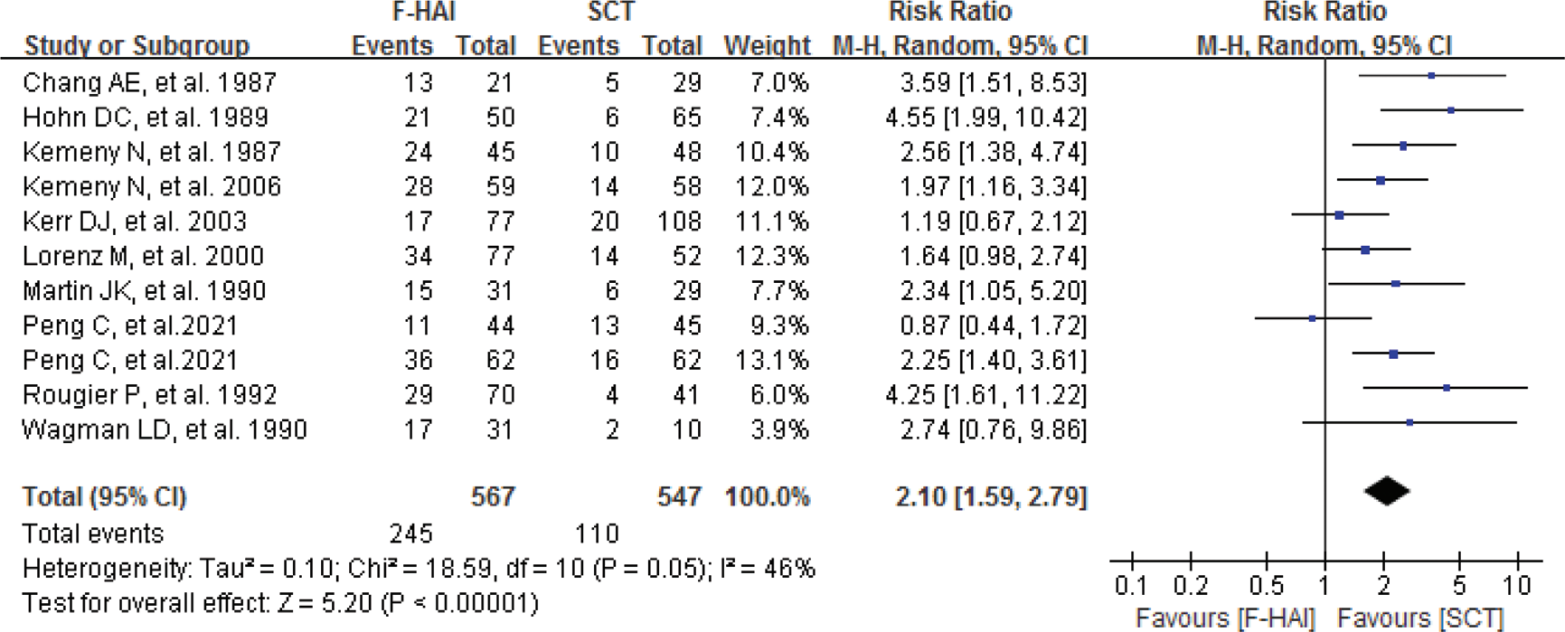
The comparison between F-HAI and SCT for CRLM regarding response rate. CRLMs = colorectal liver metastases, F-HAI = fluoropyrimidine-HAI, SCT = systemic chemotherapy.

As shown in Figure 4, approximately 43.2% (245 in 567 patients) of unresectable CRLMs experienced response in the HAI arm and approximately 20.1% (110 in 547 patients) experienced response. Our pooled result showed a higher response rate in the HAI arm compared with the SCT arm, with a pooled RR of 2.10 (95%CI 1.59–2.79; *P* < .00001). The pooled analysis was performed using a random effect model because significant heterogeneity (*P*_*h*_ = .05 and *I*^2^ = 46%) was detected among the studies.

### 3.3 Comparison of OS between F-HAI and SCT

#### 3.3.1 Data based on RCTs

Eleven RCTs with 12 set data were used to analyze the impact of the HAI treatment on patients’ OS. Singularly taken, 3 studies (30%) found a significantly better median OS time in the HAI arm compared with the SCT arm, whereas in the other 7 studies (70%) no significant difference was found. Of note, in 2 of 3 positive studies, less than 66% of patients were treated with SCT in the control arm.

**Figure 5:**
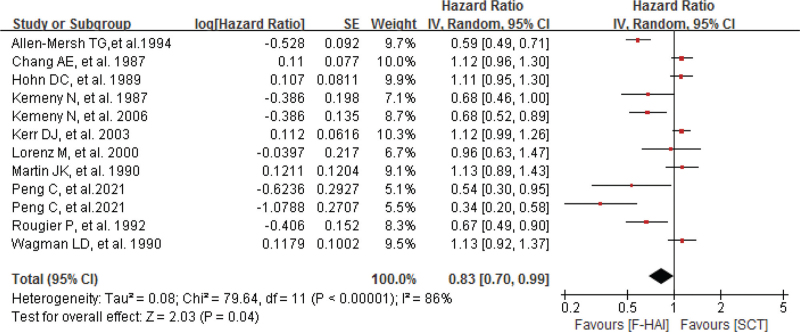
The comparison between F-HAI and SCT for CRLM regarding OS. CRLMs = colorectal liver metastases, F-HAI = fluoropyrimidine-HAI, OS = overall survival, SCT = systemic chemotherapy.

As shown in Figure 5, our pooled result showed F-HAI had a significant benefit regarding to OS, with a pooled HR of 0.83 (95% CI 0.70–0.99; *P* = .04). The pooled analysis was performed using a random-effects model because of significant heterogeneity (*P*_*h*_ < .0001 and *I*^2^ = 86%) among studies. To explore the difference of OS between the HAI arm and SCT arm, we further conducted subgroup analysis according to different characteristics such as sample size, patients treated, crossover, and study quality. However, significant results were only found in subgroups of sample size less than 99 pts (HR 1.12; 95% CI 1.01–1.25; *P* = .04) and less than 65% pts treated (HR 0.61; 95% CI 0.52–0.71; *P* < .00001). No significant difference was found in subgroups of sample size more than 99 pts (HR 0.81; 95% CI 0.64–1.03; *P* = .09); more than 65% pts treated (HR 1.02; 95% CI 0.90–1.15; *P* = .77); no crossover (HR 0.86; 95% CI 0.67–1.10; *P* = .23); crossover (HR 1.06; 95% CI 0.95–1.19; *P* = .32); high study quality (HR 1.02; 95% CI 0.87–1.18; *P* = .83), and low study quality (HR 0.81; 95% CI 0.60–1.11; *P* = .19), respectively (Table 3).

**Table 3:** The subgroup analysis of the comparison between F-HAI and SCT for CRLM regarding OS.

		Pooled results			Heterogeneity		
Outcomes	Number of pts	HR	95% CI	*P* value	*I* ^2^	*P*_*h*_ value	Analytical effect model
Sample size							
≥99 pts	1041	0.81	0.64, 1.03	.09	88%	<.0001	Random-effect model
<99 pts	174	1.12	1.01, 1.25	.04	0%	1	Fixed-effect model
Patients treated							
≥65% treated	952	1.02	0.90, 1.15	.77	62%	.01	Random-effect model
<65% treated	263	0.61	0.52, 0.71	<.0001	0%	.49	Fixed-effect model
Crossover							
No	821	0.86	0.67, 1.10	.23	90%	<.0001	Random-effect model
Yes	394	1.06	0.95, 1.19	.32	50%	.11	Fixed-effect model
Quality							
High quality	743	1.02	0.87, 1.18	.83	68%	.01	Random-effect model
Low quality	472	0.81	0.60, 1.11	.19	88%	<.0001	Random-effect model

#### 3.3.2 Data based on NRSIs

In addition, there were 5 NRSIs with 443 patients comparing OS between the HAI arm with SCT arm. Conversely, the pooled results of NRSIs showed a significant difference in OS. It was indicated that patients who received F-HAI experienced longer 1-, 2-, and 3-years OS than SCT, with pooled ORs of 2.94 (95% CI 1.21–7.13; *P* = .02); 2.31 (95% CI 1.36–3.92; *P* = .002); and 2.67 (95% CI 1.62–4.39; *P* = .001), respectively. However, a significant difference in OS was found in multivariate analysis with generic inverse variance (HR 0.39; 95% CI 0.26–0.58; *P* < .00001) but not in univariate analysis (HR 0.64; 95% CI 0.21–1.92; *P* = .43) (Table 4).

**Table 4:** The analysis of the comparison between F-HAI and SCT for CRLM regarding OS.

		Pooled results			Heterogeneity		
Outcomes	Number of pts	Estimates	95% CI	*P* value	*I* ^2^	*P*_*h*_ value	Analytical effect model
1-yr OS	385	OR 2.94	1.21, 7.13	.02	0%	.94	Fixed-effect model
3-yr OS	385	OR 2.31	1.36, 3.92	.002	8%	.34	Fixed-effect model
5-yr OS	385	OR 2.67	1.62, 4.39	.001	0%	.49	Fixed-effect model
OS (UVA)	109	HR 0.64	0.21, 1.92	.43	78%	.03	Random-effect model
OS (MVA)	211	HR 0.39	0.26, 0.58	<.00001	0%	.99	Fixed-effect model

### 3.4 Publication bias

**Figure 6:**
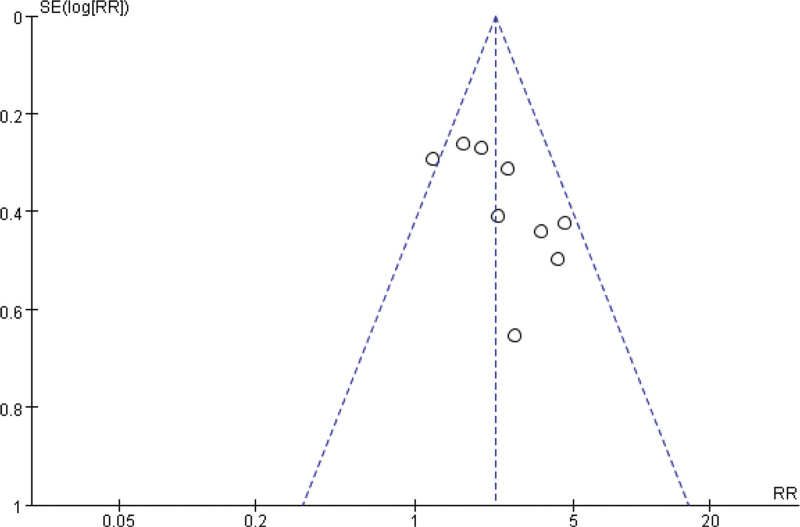
Funnel plot for detecting publication bias.

Begg funnel plot was generated to assess publication bias in the included studies. As shown in Figure 6, the plots displayed no obvious asymmetry and showed no clear evidence of publication.

## 4 Discussion

The unique differential blood supply of the liver (portal vein > healthy parenchyma; hepatic artery > metastatic disease) combined with the first-passage effect of drugs directly delivered into the liver (which allows administering higher dose of chemotherapeutics as compared to SCT) represent the rationale of a loco-regional treatment such as HAI chemotherapy for patients with unresectable CRC metastatic disease confined to the liver.^[31,32]^

FUDR (a pyrimidine antimetabolite transformed into 5-fluorouracil in the liver) is the preferred agent for HAI because of its short half-life and high rate (>90%) of hepatic extraction (a major advantage over 5-fluorouracil, whose extraction rate is <50%), leading to a 100- to 400-fold ratio of hepatic-to-systemic drug exposure.^[31]^ This allows yielding drug concentrations in the diseased organ much higher than those achievable with SCT without incurring systemic toxicity. Liver damage (mainly biliary sclerosis) is the dose-limiting toxicity, which has been reduced with the use of dexamethasone as part of the treatment, whereas catheter displacement or occlusion remains the most frequently reported complication (Allen 2005). Although randomized and non-randomized clinical trials have demonstrated that fluoropyrimidine-HAI can be followed by significantly higher tumor response rates as compared to fluoropyrimidine-based SCT, the impact of this loco-regional treatment on patients’ OS is unclear. Despite 11 RCTs and several observational studies, the therapeutic impact of fluoropyrimidine-HAI is still a matter of debate, and some oncologists/surgeons continue to propose patients this loco-regional treatment for the management of unresectable liver confined CRC metastatic disease.^[32–36]^

In the present meta-analysis, we considered for the first time all 11 RCTs so far performed. The findings of our work indicate that fluoropyrimidine-based HAI provides a significant tumor response advantage when compared with fluoropyrimidine-based SCT (43.2% vs 20.1%, respectively). However, this benefit is of no clinical value because modern SCT regimens (ie, those combining 5-fluorouracil with oxaliplatin or irinotecan) can obtain tumor response rates similar to or even higher than those observed with fluoropyrimidine-based HAI.^[3]^ More importantly, this meta-analysis shows that fluoropyrimidine-HAI is not associated with an OS advantage when compared with fluoropyrimidine-based SCT. Though the pooled results of NRSIs showed better OS of fluoropyrimidine-HAI, the sample size was really small which may lead to any bias.

As regards the overall effect of HAI on survival, our results (ie, HAI does not improve patients’ survival as compared to SCT) are in contrast with 2 previous meta-analyses published more than 10 years ago. These meta-analyses considered 6 of 7 RCTs available at that time and demonstrated a small but significant survival advantage of HAI over SCT.^[4,5]^ In their pooled analysis based on individual patient data,^[5]^ the investigators of the Meta-Analysis Group in Cancer did not include the study by Hohn et al, 1989 (n = 143) due to not better specified “logistic reasons.”^[19]^ They found that HAI was associated with a 27% risk reduction of death (*P* = .0009); at subgroup analysis, this survival advantage disappeared when they removed from meta-analysis the 2 RCT in which less than 66% of patients received SCT in the control arm.

Our finding may be particularly discouraged by some deficiencies. First, the present meta-analysis includes 2 RCT with a low percentage (<66%) of patients receiving SCT in the control arm, which should be regarded as a potential bias in favor of HAI: therefore, the negative results of the present meta-analysis have been obtained in spite of the potential bias in favor of HAI. Second, as compared to the results reported using HAI, similar or even better median OS times are reported after modern SCT regimens, which are often tested in series including patients with hepatic as well as extra-hepatic metastatic disease (greater tumor burden indicates worse prognosis): accordingly, the negative findings of the present meta-analysis have been obtained considering as a control group patients treated with an “old fashion” SCT whose results are outperformed by more modern regimens. Finally, subgroup analyses considering high-quality RCT (ie, those with adequate sample size, with more than 66% of patients receiving the assigned treatment in both arms, or without crossover to HAI) confirmed the lack of impact on survival of fluoropyrimidine-HAI, further strengthening the evidence that this loco-regional treatment does not add any significant survival advantage over SCT (Supplemental Digital Content "AMSTAR-2," .).

This meta-analysis demonstrated a beneficial effect of HAI in terms of OS based on all 11 RCTs so far carried out, which enrolled more than 1,700 patients. In the light of the improved efficacy of modern SCT regimens, as compared to the SCT regimens compared to HAI in the 11 RCTs, the findings of the present meta-analysis are further strengthened and must be taken into consideration in the therapeutic management of these patients to provide them with the best treatment.

Although fluoropyrimidine-HAI alone should be abandoned for the treatment of CRC liver metastatic disease not amenable to surgical excision, HAI might still be exploited for the loco-regional delivery of novel anticancer agents or drug combinations.^[37]^ In particular, HAI should be associated with modern SCT, as already under clinical evaluation for balancing the major and intrinsic limit of loco-regional treatments, which is the lack of therapeutic control over clinically occult extra-hepatic minimal residual disease.^[38,39]^

## 5 Conclusion

Our results indicated that the F-HAI regimen had a greater tumor response rate and survival advantage than SCT for patients with unresectable CRLMs. Future propensity score-matched analyses with a large sample size should be conducted to support the evidence of our results based on RCTs and NRSIs.

## Author contributions

**Conceptualization:** Jianmeng Zhao.

**Data curation:** Jianmeng Zhao, Tao Liu.

**Formal analysis:** Jianmeng Zhao, Tao Liu.

**Methodology:** Hongqing Shan, Ke Cong.

**Software:** Tao Liu, Jinzhe Chang, Hongqing Shan, Ke Cong.

**Supervision:** Jinzhe Chang.

**Writing – original draft:** Jianmeng Zhao, Yuenan Zheng, Jinzhe Chang, Ke Cong.

**Writing – review & editing:** Jianmeng Zhao, Yuenan Zheng, Hongqing Shan, Ke Cong.

## Supplementary Material

Supplemental Digital Content
